# What should the African health workforce know about disasters? Proposed competencies for strengthening public health disaster risk management education in Africa

**DOI:** 10.1186/s12909-018-1163-9

**Published:** 2018-04-02

**Authors:** Olushayo Olu, Abdulmumini Usman, Kalula Kalambay, Stella Anyangwe, Kuku Voyi, Christopher Garimoi Orach, Aklilu Azazh, Mala Ali Mapatano, Ngoy Nsenga, Lucien Manga, Solomon Woldetsadik, Francois Nguessan, Angela Benson

**Affiliations:** 1 World Health Organization, PO Box 1324, Kigali, Rwanda; 2 WHO Regional Office for Africa (AFRO), Brazzaville, Republic of Congo; 3 International Public Health Disaster Risk Management Consultant, Gatineau, Canada; 4 School of Health Systems and Public Health, University of Pretoria, Pretoria, South Africa; 5 School of Public Health, Makerere University, Kampala, Uganda; 6 Faculty of Medicine, University of Addis Ababa, Addis Ababa, Ethiopia; 7 School of Public Health, Faculty of Medicine, University of Kinshasa, Kinshasa, Democratic Republic of the Congo; 8 WHO Country Office, Bamako, Mali; 9 WHO Emergency Support Hub, Nairobi, Kenya; 10 International Public Health and Disaster Risk Management Consultant, Monrovia, Liberia

**Keywords:** Public health, Disaster risk management, Education, Core competencies, Training curricula, Health workforce, Africa

## Abstract

**Background:**

As part of efforts to implement the human resources capacity building component of the African Regional Strategy on Disaster Risk Management (DRM) for the health sector, the African Regional Office of the World Health Organization, in collaboration with selected African public health training institutions, followed a multistage process to develop core competencies and curricula for training the African health workforce in public health DRM. In this article, we describe the methods used to develop the competencies, present the identified competencies and training curricula, and propose recommendations for their integration into the public health education curricula of African member states.

**Methods:**

We conducted a pilot research using mixed methods approaches to develop and test the applicability and feasibility of a public health disaster risk management curriculum for training the African health workforce.

**Results:**

We identified 14 core competencies and 45 sub-competencies/training units grouped into six thematic areas: 1) introduction to DRM; 2) operational effectiveness; 3) effective leadership; 4) preparedness and risk reduction; 5) emergency response and 6) post-disaster health system recovery. These were defined as the skills and knowledge that African health care workers should possess to effectively participate in health DRM activities. To suit the needs of various categories of African health care workers, three levels of training courses are proposed: basic, intermediate, and advanced. The pilot test of the basic course among a cohort of public health practitioners in South Africa demonstrated their relevance.

**Conclusions:**

These competencies compare favourably to the findings of other studies that have assessed public health DRM competencies. They could provide a framework for scaling up the capacity development of African healthcare workers in the area of public health DRM; however further validation of the competencies is required through additional pilot courses and follow up of the trainees to demonstrate outcome and impact of the competencies and curriculum.

**Electronic supplementary material:**

The online version of this article (10.1186/s12909-018-1163-9) contains supplementary material, which is available to authorized users.

## Background

The health system framework has been increasingly proposed as the basis for effectively implementing disaster risk management (DRM) within the realm of public health [1–3]. A health system encompasses people and organizations as well as actions; therefore, it is defined as “consisting of all organizations, people and actions whose primary intent is to promote, restore or maintain health” [4]. The health system has six building blocks namely 1) health leadership and governance, 2) health products and technologies, 3) health workforce, 4) financing of health services, 5) management of health information, 6) delivery of health services [4]. These building blocks are interrelated and work in tandem to ensure good health services provision. A resilient health system should have the capacity to effectively prepare for and respond to disasters, preserve key health functions, and recover during and after disaster strikes [5]. Such a system should guarantee good access, coverage, quality, and safety of health services [4], safeguard human life and ensure good health outcomes during and after disasters [5].

The health workforce includes any person who is involved in activities with the ultimate intention to protect and improve health; these include people working in the public and private sectors, formal and informal sectors, and at the community level [4] A strong health workforce is critical for ensuring a resilient health system [5–7], which in turn is a key element of effective public health DRM. The presence of adequate numbers of skilled health care workers (HCWs) is a key determinant of health outcomes during disasters [8]. Available literature points to weak health systems and inadequate numbers of skilled HCWs as key factors that sustained transmission of the 2014–15 Ebola outbreaks in West Africa [9, 10]. Furthermore, a World Health Organization (WHO) report identified the inadequate numbers of HCWs in the Ebola-affected countries, poor training, and a lack of required medical equipment as part of the challenges that hampered early control of the outbreak [11]. Similar challenges have also been observed during other humanitarian crises on the continent, such as the Horn of Africa and Sahel drought crises as well as the ongoing Yellow Fever and Zika virus outbreaks [12, 13]. These events highlight the need for well-trained and skilled health care workers for public health DRM.

In 2012, the sixty-second session of the WHO Regional Committee for Africa adopted (through resolution AFR/RC62/R1) and is currently implementing a 10-year regional strategy on DRM for the health sector [14]. This strategy aims to improve the capacity of the health sector and to build the institutional capacity of WHO African Region Member States^1^ to manage disasters. Furthermore, it intends to improve individual HCW competencies for public health DRM, so as to strengthen resilience in the community and health sector. Well-trained and competent health workers were identified as a critical requirement for effective implementation of the strategy, and thus resolution AFR/RC62/R1 includes a clause requesting WHO to “support national capacity building for DRM” [15].

As part of the efforts to implement the human resources capacity building component of the strategy, the WHO African Regional Office (WHO/AFRO) conducted an unpublished desk review of existing emergency public health training programmes in the African Region.^2^ The review showed that there were few available public health emergency courses on the continent, most of which were focused on response to public health disasters especially epidemics and lack critical aspects of public health disaster risk reduction, preparedness and post-disaster health system recovery. Furthermore, the review revealed that the contents of many of the available courses varied and did not include contemporary humanitarian topics, such as the international humanitarian reforms, transformative agenda, legal framework for humanitarian response, and emergency response operations management. This emphasized the need for a comprehensive and harmonized public health DRM training package for African HCWs.

To address this gap, WHO/AFRO, in collaboration with selected African public health training institutions, conducted a multistage pilot study to develop and test comprehensive core competencies and curricula for training the African health workforce in public health DRM. In this article, we present the competencies and training curricula that were identified, describe the stages of their development, and propose recommendations for their completion and integration into the public health education curricula of African public health training institutions. The general objective of the article is to provide information and opportunities that could be used to improve health DRM training in Africa.

## Methods

We conducted a pilot research to develop and test a public health disaster risk management curriculum for training the African health workforce on DRM. We used mixed methods approaches including literature review to better understand the public health disaster risk management training needs and the associated knowledge and skills required by the African health workforce; qualitative research methodology to develop the core competencies and curriculum; knowledge translation and quantitative research methodology to test the applicability and feasibility of the competencies and curriculum.

We obtained curriculum development terminologies via online searches of Google search engine using the term “curriculum development and terminologies”. The search yielded several documents which we reviewed. We selected three of the documents for in-depth review.^3^ The review of the three documents yielded over 30 terminologies out of which we selected four key ones which were relevant to our purpose. These are competency, which was defined as a cluster of related knowledge, skills, and attitudes that enable an individual or organization to perform their core functions well [16]. Learning objectives were defined as a description of what students should know or be able to do at the end of a course that they could not do before the course [17]. We also defined a curriculum as the planned interaction of student with instructional content, materials, resources, and processes for evaluating the attainment of educational objectives [18], and training module was defined as a standardized or self-contained segment that constitutes an educational course or training programme [19].

We implemented the curriculum development from 2009 to 2013, using a multistage consultative and qualitative process. A team of African emergency public health academicians and practitioners was assembled to: 1) conduct an in-depth review of existing emergency public health training programmes in the African Region; 2) identify knowledge, skills sets, and core competencies required by African HCWs to effectively engage in DRM; 3) develop a generic training curriculum for health DRM that is suited to the region; and 4) agree on the modalities for pilot testing the courses. Participants from the schools of public health of six African universities^4^ as well as emergency public health practitioners from Ministries of Health, the US Centers for Disease Control and Prevention, and the three levels of the WHO participated in the process.

The first stage involved a desk review of the content of available in-service, basic, and specialized training programmes in public health DRM across the African Region as well as agreement on a road map for development of health DRM curricula for Africa and the methodology for identification of the core competencies. The second stage involved the establishment of an up-to-date inventory of existing public health DRM training programmes, establishment of partnerships between training institutions and relevant stakeholders to enhance programme quality and continuity, and production of a framework for standardizing training packages with the aim of addressing the continuum of the disaster management cycle at all levels.

In the third stage, we reviewed and listed the knowledge and skills required to effectively implement the regional health DRM strategy through compilation and desk review of the existing literature on health DRM and the associated skill sets and core competencies. At this stage, we organized a consultative meeting to discuss and validate the identified knowledge and skill sets. During the meeting, participating academic institutions presented the health DRM competencies used to develop the public health training curricula of their institutions, which were then compared with the identified list of competencies to ensure that the list was inclusive. A predeveloped template was used to group the knowledge and skills into competencies and sub-competencies, which were then categorized into thematic areas. The learning objectives for each competency were identified and curricula developed for basic, intermediate, and advanced health DRM courses. Finally, all the products of the consultative meeting were endorsed by the experts present who thoroughly reviewed and compared them to what existed in the peer-reviewed literature. At the fourth stage, the final products were sent for peer review to the relevant programmes of WHO/AFRO, selected ministries of health, and public health training institutions.

In the fifth and final stage, we reviewed the core competencies, learning objectives, and course curricula based on feedback received from the peer review process; we also developed the training units and modules for all core competencies, compiled the work done thus far, and developed a template for writing a guideline/framework for health sector DRM capacity building.

We conducted a pilot test of the basic public health DRM training module at one of the participating training institutions (the University of Pretoria) in conjunction with the South African National Defence Forces (SANDF) and WHO/AFRO in March 2015, to test the applicability and feasibility of the core competencies and curricula. We used quantitative methods (pre- and post-tests) to assess the performances of all the participants who attended the pilot course. Similarly, all participants evaluated the relevance of the course and the competencies addressed in it.

## Results

### Core competencies for public health DRM

We identified 14 core competencies and 45 sub-competencies/training units, grouped into six thematic areas, as the skills and knowledge that African health care workers should possess to be able to effectively engage in public health DRM (Table 1). These six thematic areas are as follows: 1) introduction to DRM; 2) operational effectiveness; 3) effective leadership; 4) preparedness and risk reduction; 5) emergency response; 6) post-emergency health system recovery.

1. **Introduction to DRM:** All health care professionals engaged in health DRM should understand the basic principles of DRM. This thematic area has four core competencies that focus on understanding the principles of public health and disasters, principles of human rights and ethics in disasters, basic epidemiology and data management, and effective communication during disasters. This thematic area is further subdivided into 12 sub-competencies/training units (Table 1). The 14 core competencies, their training units, and learning objectives are outlined in Additional file 1.
2. **Operational effectiveness:** Acquisition of soft skills in the areas of resource mobilization and management, basic logistics management, and application of staff safety and security measures were considered critical elements of a sound public health DRM curriculum; therefore, health care workers should have the necessary skills and knowledge in these areas. This thematic area has four sub-competencies/training units (Table 1).
3. **Effective leadership:** This thematic area focuses on skills and knowledge associated with leadership, management, coordination, supervision, monitoring, and evaluation of public health DRM programmes and is subdivided into seven sub-competencies/training units (Table 1).
4. **Preparedness and risk reduction:** The ability to conduct various types of assessments, plan and implement public health preventive and mitigation measures, and to establish preparedness interventions at the health facility and community levels were identified as critical competencies that health workers should possess to effectively reduce the risk of and to prepare for disasters. This thematic area is subdivided into 12 sub-competencies/training units (Table 1).
5. **Emergency response:** The ability to apply DRM principles and practices for the health response to disasters was identified as a critical competency that health workers should possess to effectively respond to disasters. This thematic area is subdivided into 6 sub-competencies/training units (Table 1).
6. **Post-emergency health system recovery:** The ability to apply DRM principles and practices to plan and implement post-emergency health system and population recovery interventions, were identified as critical competencies that health workers should possess to effectively recover from disasters. This thematic area is subdivided into 4 sub-competencies/training units (Table 1).

**Table 1:** Thematic areas and public health emergency core competencies for training African health workers

Thematic area	Core Competency	Training units
Introduction to DRM	1. Demonstrate knowledge of public health principles and practices for Disaster Risk Management	1.1 Disaster risk management concepts 1.2 Public health consequences of disasters 1.3 Context: political, social and economic environment
	2. Demonstrate knowledge of basic epidemiological methods and data management	2.1 Basic epidemiology 2.2 Data analysis and management
	3. Demonstrate the ability to communicate effectively in DRM	3.1 Key principles of communication 3.2 Risk communication 3.3 Operational communication(any communication that is not with the media and public)
	4. Demonstrate the knowledge of principles of legal considerations, human rights and ethics in dealing with DRM	4.1 Ethics 4.2 Human rights 4.3 International humanitarian law 4.4 International health regulations
Operational Effectiveness	5. Demonstrate ability to identify, mobilise and manage resources	5.1 Resource mobilization
	6. Demonstrate the ability to apply logistics management	6.1 Logistics management
	7. Demonstrate the ability to apply measures of safety and security	7.1 Basic security in the field 7.2 Protection and Family safety
Effective Leadership	8. Demonstrate effective leadership, teamwork and management skills required for DRM	8.1 Principles of leadership and management 8.2 Leadership 8.3 Management 8.4 Coordination
	9. Demonstrate knowledge about the monitoring and evaluation cycle	9.1 Key principles 9.2 Monitoring 9.3 Evaluation
Preparedness and Risk Reduction	10. Demonstrate the ability to conduct capacity assessments	10.1 Key principles of capacity assessments 10.2 Risk assessments 10.3 Needs assessments
	11. Demonstrate the ability to plan and implement preventive and mitigation activities	11.1 Key principles of prevention and mitigation 11.2 Risk reduction 11.3 Mitigation
	12. Demonstrate the ability to plan and implement emergency preparedness at community and health facility levels	12.1 Key principles of disaster preparedness 12.2 Planning 12.3 Early warning 12.4 Surge capacity 12.5 Training 12.6 Exercise management
Emergency Response	13. Demonstrate ability to apply DRM principles and practices for the health response to disasters and public health emergencies	13.1 Key principles of emergency health response 13.2 Health assessment 13.3 Mass casualty management and emergency health services 13.4 Incident management 13.5 Public health programmes in emergencies 13.6 Planning
Post-emergency health system recovery	14. Demonstrate ability to plan and implement health system and population recovery.	14.1 Key principles of post-disaster health system recovery 14.2 Recovery needs assessments 14.3 Recovery strategy and planning 14.4 Recovery programme implementation

### Training curricula for public health DRM

Based on the 14 core competencies, we identified three levels of training to suit the needs of various categories of African HCWs, namely, basic, intermediate, and advanced training in public health DRM (Table 2). The delivery and assessment methods for these courses include as modules in undergraduate studies, post-graduate diploma or public health masters courses, or as stand-alone programmes (Table 3). The curricula for basic, intermediate, and advanced training in health DRM are presented in detailed in Additional files 2 and 3).

**Table 2:** Proposed health DRM training curricula for African health care workers

Curriculum	Objectives	Target audience	Admission criteria	Competencies addressed	Proposed duration
Basic	The course will enable participants to define and apply basic knowledge about DRM to fulfil their role effectively in DRM	a) Health worker at the district level/or equivalent. b) Staff of humanitarian agencies c) Personnel who contribute to Health DRM from other sectors a) Persons interested in following a track in DRM as a carrier (academic, professional)	Persons eligible for this course must have at least a basic certificate/diploma (nursing, paramedical and/or medicine/dentistry, environmental health, veterinary science or social sciences)	Introduction to the principles of all the core competencies except for competency 5 and 6	48 h (approximately 6 working days)
Intermediate	The course will enable participants to apply knowledge of, and skills in health DRM to fulfil their role as effective sub-national managers in DRM and acquire the knowledge, attitude and skills to contribute to building country resilience	This course is applicable to mid-level health care personnel working in Health DRM at sub-national and national levels.	This course is applicable to participants with the following qualifications; a minimum of Bachelor’s degree in health or social sciences, with at least 1 year experience in health DRM or a basic diploma in health or social work, with the certificate of the basic course in Health DRM, or over 3 years’ experience in DRM.	All 14 core competencies are fully addressed in this course	134 h (approximately 14 working days)
Advanced	The course will enable participants to apply advanced management and leadership knowledge and skills about DRM to fulfil their role at national levels effectively and to have the knowledge, attitude and skills to contribute to building country resilience	This course is applicable to all personnel working in Health DRM at regional and national levels, and those intending to lead and manage Health DRM at national level.	This course is applicable to participants with the following qualifications; ▪ A basic or Master’s degree in health or social sciences, with at least 3 years’ experience in health DRM, PLUS ▪ The certificate in the Intermediate course in Health DRM. ▪ Attendance of the intermediate course is a prerequisite for being selected for this course	This course mainly involves an internship period of about 8 weeks, during which participants will undergo practical field placements. 1 week will be spent on orientation and briefing by the training institution, 6 weeks on field work, and 1 week for report writing, debriefing and oral defence of the report. The internship exercise involves application of the knowledge, attitude and skills related to the DRM cycle. Therefore, the intern will rotate through either: ▪ An emergency situation, preferably at the local level ▪ Field work on preparedness for a disaster	14 working days (for the intermediate) plus 8 weeks for field placement

**Table 3:** Delivery options for basic, intermediate and advanced public health DRM courses in Africa

Course	Delivery method	Assessment	Certification
Basic course in DRM	Incorporated into modules for undergraduate studies in medicine, other health sciences and public health.	Based on university criteria for assessing the undergraduate course into which it is incorporated.	Incorporated into undergraduate degree award.
	Stand-alone course	Written examination	^a^Certificate of competence of the participating University or training institution.
Intermediate course in DRM	Incorporated as a module into Post-graduate diploma programmes which are relevant to DRM.	Based on university criteria for assessing the Postgraduate diploma programme into which it is incorporated.	Incorporated into Postgraduate diploma
	Incorporated as a module into Master’s programmes which are relevant to DRM, such as MPH, MSc etc.	Based on university criteria for assessing the Master’s degree programme into which it is incorporated	Incorporated into Master’s degree.
	Stand-alone course	Written examination	Certificate of competence of the participating University or training institution.
Advanced course in DRM	As the health DRM track of health-related Master’s degree programmes, such as MPH or MSc	The assessment will be through observation of the field supervisors (20%), written field report (50%), verbal defence of the report (30%). Observation by the field supervisor will be guided by a questionnaire, and the field supervisor may be a member of the oral defence committee.	

^a^The participant will have to do a written exam to be awarded the certificate of competence

### Piloting the basic public health DRM course

Twenty-six participants (24 SANDF officers or soldiers and two civilians) attended the pilot basic health DRM training course, which was administered by five facilitators from the University of Pretoria School of Health Systems and Public Health (SHSPH) and WHO/AFRO. Thirteen lectures and three group work sessions were delivered over the 5-day pilot course. During the group work sessions, trainees used actual examples of disasters such as the Ebola outbreak in Sierra Leone, flooding in northern Namibia, cholera outbreak in Zimbabwe, and the armed conflict in northern Uganda, to simulate and practise the principles of disaster preparedness, risk reduction, response, and recovery. The course covered basic principles of 12 out of the 14 competencies namely: 1) public health principles and practices for DRM; 2) basic epidemiology; 3) risk communication; 4) ethics, human rights, international humanitarian law, and international health regulations; 5) safety and security; 6) leadership and management; 7) monitoring and evaluation; 8) capacity assessment; 9) prevention and mitigation; 10) preparedness; 11) response; and 12) recovery.

The average scores for the pre- and post-tests were 51.5% and 59.2%, respectively. All participants agreed that the course was very appropriate and that their expectations had been met. Nearly all trainees (except one) agreed that the 12 core competencies covered were all relevant to the course and to their public health DRM work. Participants felt that the data management, disaster preparedness, risk reduction, and response sessions were intensive and that more time should be allocated to these in future courses.

## Discussion

The lessons learnt from recent public health events of international concern in Africa have revealed the fragility of the continent’s health systems [10, 20] and the critical role of HCWs in the public health emergency preparedness, response and recovery framework [21, 22]. These events have demonstrated that competent health workers are needed not only to lead public health services delivery, support planning, supervision, coordination, and evaluation of all aspects of public health disaster management [23–26] but also to prevent the health workforce from being infected or affected by disasters. Significant attention to and investment in the health workforce are therefore critical to ensure resilient health systems that can effectively prepare for, respond to, and recover from disasters [27, 28] Addressing this challenge requires increased numbers as well as improvement in the knowledge and skills of health workers [27, 29, 30]. To the best of our knowledge, there is no comprehensive set of localized competencies to guide these aspects in Africa; therefore, the findings of this study contribute to addressing this perceived gap.

We identified 14 core competencies in six thematic areas, which African HCWs should acquire to be able to effectively engage in DRM. These competencies could serve as practical guidance for African public health training institutions; ministries of health, education and disaster management; agencies of the United Nations; and national and international nongovernmental organizations, to develop public health DRM educational materials and training programmes for a broad-based category of public health students and workers alike. We propose acquisition of these competencies at three levels, namely, basic, intermediate and advanced. All front-line health workers at district level, junior staff of humanitarian agencies, and personnel from other sectors such as water, sanitation, shelter, and education who contribute to health DRM, should possess and be able to apply basic health DRM skills to manage public health disaster situations at the local level. All mid-level HCWs at provincial and national levels should have and be able to apply intermediate skills and knowledge of all 14 core competencies; HCWs who have direct responsibility for health DRM at national and regional levels should possess and be able to apply advanced health DRM skills and knowledge. We recommend acquisition of the knowledge, skills, and attitudes associated with these competencies through different methodologies. Knowledge could be gained through reading, didactic teaching, projects, assignments and experience sharing while skills and attitudes could be acquired through practical field experience and simulation exercises. These acquisition methodologies should be adapted to suit the local training needs and context.

These competencies, together with the associated curricula and training programmes, are proposed as the minimum requirements for all African HCWs that are involved in public health disaster management. However, we believe that there should be opportunities for African HCWs to attend specialized courses, such as in health disaster risk reduction, mass casualty management, and post-disaster health system recovery, so as to acquire more in-depth knowledge of the core competencies. We identified potential challenges that would need to be addressed to ensure the smooth rollout of these competencies. These include the tendency for courses to focus on didactic teaching methods as opposed to a more practical approach, which might limit field experience among students. Other challenges include the shortage of experienced trainers at the initial stages of rollout of the competencies and curricula, insufficient time allocated for the training programmes, and shortages of funding for public health training institutions to introduce the core competencies into their training programmes. Additional challenges include inadequate financial support for African health workers to participate in training programmes, resistance by African training institutions to introduce the core competencies, and a lack of modalities for uniform accreditation of the courses across African countries and training institutions.

Effective implementation and sustainability of these core competencies would require their integration into the broader framework of the Global Health Workforce Alliance, existing public health education, the DRM policies of African member states, and ongoing efforts to strengthen the global emergency health workforce and health security. Therefore, a number of success factors such as high-level political commitment; government involvement and financial investment; good strategic planning for public health DRM workforce education; effective management, leadership, and information; and supervision, monitoring, and evaluation systems are critical [7, 31]. In this regard, we propose a number of principles that should guide the use of these core competencies to develop public health DRM training programmes and courses.

First, courses should be sustainable, affordable, and accessible and they should be based on training needs that have been identified through training needs assessment. Second, sequencing of and time allocated to the training units and modules should be logical and realistic, to ensure coherence in the course delivery. Third, the principles of right to health, human rights, equity, social justice, and humanitarian assistance should be mainstreamed into the health DRM training courses and programmes. Fourth, the courses should be regularly monitored and evaluated, to ensure that they are of high quality and that they not only meet their objectives and the learning needs of trainees but also the skill needs of African member states. Fifth, in view of the multisectoral nature of DRM, the planning, delivery, and evaluation of training courses should involve experts from other relevant sectors, clusters, organizations and academic institutions as trainers and participants. Lastly, strong advocacy and formation of partnerships between African ministries of health, education, and disaster management, academic institutions, and international and local organizations are required, to ensure adequate funding, development of sustainable funding modalities, availability of the necessary capacities and management systems for public health DRM training programmes in Africa. This would also support consensus building on certification modalities for the courses.

These core competencies should be adapted to the local context, and the resulting training courses should be as practical and field-based as possible. The successful implementation of these core competencies requires strong advocacy at the level of African health, education, and disaster ministries to ensure the availability of appropriate inputs such as funds, human resources, and training materials. We believe that African public health training institutions have the required environment to introduce these competencies in their curricula; these institutions therefore represent one of the greatest assets in scaling up public health DRM capacity building on the continent.

### Study limitations

This study has three main limitations. First, not all members of the team participated in all stages of the consultative process, which were spread over a 3-year period; this could have resulted in memory lapses that might have biased the study findings. Second, the process involved selection of only a small number of African public health training institutions, academicians, and public health DRM practitioners, whose views on health DRM core competencies may not be representative of the views of all African public health experts and training institutions. We mitigated these limitations through peer review of the final outcomes of the consultative process by other African public health academicians and health DRM practitioners in the African Region. Third, the sample size of the cohort of people who participated in piloting of the basic course is small and may not be significant. Furthermore, we were unable to follow up this cohort of people to determine whether they were able to successfully apply the skills acquired during the pilot and if this had an impact on their future public health DRM work thus further pilot testing and validation of all three courses are required.

## Conclusions

Scaling up the capacity development of African HCWs in the areas of public health DRM is critical to ensure resilient health systems. This requires a structured and uniformed approach and guidelines that can be adapted across the continent. The core competencies and sub-competencies identified in this article provide opportunities for addressing this task. These competencies compare favourably to the findings of other studies that have assessed public health DRM competencies [32–35]. The pilot test of the basic course demonstrated the relevance of the core competencies and that their use could result in improving the understanding of public health DRM among participants. However, in view of the small sample size of the pilot course, further validation of these competencies through additional pilot courses and follow up of the trainees is required to establish their outcome and impact on public health DRM. Moving forward, strong advocacy as well as government ownership and support of the public health DRM community, and establishment of African public health DRM training institution networks, are required to scale up public health DRM capacity building in Africa.

To effectively roll out these competencies and curricula across the continent, we put forward four main recommendations. First, WHO/AFRO in collaboration with participating training institutions, should conduct more pilot tests of the basic course and finalize those of the intermediate and advanced courses, develop the learning materials for all three courses, and create awareness about these core competencies and curricula among African public health training institutions and ministries of health, education, and disaster management. Second, WHO/AFRO should liaise with ministries of education and health of its member states to discuss the modalities for their accreditation. Furthermore, the organization should work with these ministries to conduct training of trainers so as to establish a critical mass of trainers who could support initial courses. Third, African public health training institutions should conduct assessments of their emergency public health training programmes, using the above-mentioned competencies as a baseline, to improve their planning and curriculum development for public health DRM training programmes where necessary. Lastly, WHO/AFRO in collaboration with training institutions and ministries of health should develop a monitoring and evaluation framework to ensure the quality of public health DRM training programmes across the African Region [31]. This should include development of relevant process, outcome, and impact indicators that should be used to monitor and evaluate the quality and impact of public health DRM training programmes, trainees, and trainers in the region.

## Additional files

Additional file 1: Health DRM core competencies, sub-competencies and learning objectives. (DOCX 39 kb)

Additional file 2: Curriculum for basic, intermediate and advanced training in health DRM. (DOCX 38 kb)

Additional file 3: Proposed public health DRM training modules. (DOCX 23 kb)

## Abbreviations

DRM: Disaster risk management
HCW: Health care worker
SANDF: South African National Defence Forces
WHO/AFRO: African Regional Office of the World Health Organization

## Footnotes

1 Algeria, Angola, Benin, Botswana, Burkina Faso, Burundi, Cabo Verde, Cameroon, Central Africa Republic, Chad, Comoros, Republic of Congo, Cote d’Ivoire, Democratic Republic of the Congo, Equatorial Guinea, Eritrea, Ethiopia, Gabon, The Gambia, Ghana, Guinea, Guinea Bissau, Kenya, Lesotho, Liberia, Madagascar, Malawi, Mali, Mauritania, Mauritius, Mozambique, Namibia, Niger, Nigeria, Rwanda, São Tomé and Principe, Senegal, Seychelles, Sierra Leone, South Africa, Republic of South Sudan, Swaziland, Togo, Uganda, United Republic of Tanzania, Zambia, and Zimbabwe.

2 The reviewed courses include: Health Emergency in Large Populations (HELP); Public Health in Complex Emergencies (PHCE); Field Epidemiology and Laboratory Training Programme (FELTP), Analysing Disrupted Health Systems (ADHS); Public Health Pre-Deployment Course (PHPD), Health Cluster Coordination Training (HCCT).

3 The selected documents are: 1) UNESCO International Bureau of Education Curriculum Terminology; 2) Columbia University School of Nursing Competency-to-Curriculum Toolkit; 3) British Columbia Ministry of Education Glossary of Curriculum Terms.

4 1) University of Pretoria, South Africa; 2) University of Kinshasa DRC; 3) Addis Ababa University, Ethiopia; 4) Makerere University, Uganda; 5) Maputo University, Mozambique; and 6) Institute of Public Health, Benin.
