# Supplementary material for: What should the African health workforce know about disasters? Proposed competencies for strengthening public health disaster risk management education in Africa
Source: BMC Med Educ. 2018 Apr 2;18:60. doi: 10.1186/s12909-018-1163-9 (PMC5879558; doi:10.1186/s12909-018-1163-9)
Supplement: Supplementary file 1 — Health DRM core competencies, sub-competencies and learning objectives. (DOCX 39 kb) [file 12909_2018_1163_MOESM1_ESM.docx]

**Additional file 1: Health DRM core competencies, sub-competencies and learning objectives**

|  | **Core competencies** |  | **Units** | **Learning objectives** | **Main points** |
| --- | --- | --- | --- | --- | --- |
| 1 | Demonstrate knowledge of public health principles and practices for Disaster Risk Management | A | DISASTER RISK MANAGEMENT CONCEPTS | 1. Define key terminologies 2. Describe the types of hazard 3. Explain All-Hazard/Whole Health approach to DRM 4. Describe the disaster risk management cycle | 1. Basic terms and concepts used in risk management 2. Whole health and one-health approach 3. DRM cycles |
|  |  | B | PUBLIC HEALTH CONSEQUENCES OF DISASTERS | 1. Describe the public health consequences of different hazards 2. Describe and discuss routine PH programmes and health systems contribution to DRM 3. Describe possible risks and vulnerabilities associated with different hazards | 1. Elements Routine Public Health Programs 2. Public health Risk factors and vulnerabilities 3. Public health impact of hazards on communities and health systems |
|  |  | C | CONTEXT: POLITICAL,SOCIAL AND ECONOMIC ENVIRONMENT | 1. Explain the roles and responsibilities of different agencies and organisations from health and other sectors involved in DRM locally and internationally 2. Describe the political, social, economic, health and security context of disasters | 1. Main actors involved and understand their roles 2. Contextual analysis political, social, economic aspects of the crisis |
| 2 | Demonstrate knowledge of basic epidemiological methods and data management | A | BASIC EPIDEMIOLOGY | 1. Discuss basic principles of epidemiology and its application in DRM 2. Explain key epidemiological methods 3. Discuss methods for collection of data 4. Describe significance of data collection during each phase of a disaster 5. Demonstrate ability to craft basic public health report | 1. Key terminologies in Epidemiology 2. Common tools and methods in epidemiology 3. Data collection methods in disasters 4. Basic analysis and interpretation |
|  |  | B | DATA ANALYSIS AND MANAGEMENT | 1. Describe basic data analysis and interpretation including report writing in DRM context 2. Summarize data using the appropriate tools in public health data management 3. Discuss the concept and use of e-health technology within the context of DRM 4. Participate in research activities and their application | 1. Basics of data analysis 2. Data interpretation 3. Report writing 4. Concepts and use of e health technology 5. Basics of research in health emergency management |
| 3 | Demonstrate the ability to communicate effectively in DRM | A | KEY PRINCIPLES | 1. Describe the principles of effective communication Define the various channels of communication 2. Discuss key considerations in dealing with the different players in DRM context | 1. Principles of effective communication 2. Communication channels in disaster situations 3. Basics of collaborating with the media, donors, public, decision-makers 4. Basics of communication strategies and application in DRM situations |
|  |  | B | RISK COMMUNICATION | 1. Discuss the principles of crisis and emergency risk communication in a disaster or public health emergency 2. Identify strategies for appropriate sharing of information in a disaster or public health emergency 3. Identify cultural and political issues and challenges in the development and dissemination of risk communication in DRM | - Principles of effective risk communication in DRM situations - Crisis and emergency risk communication - Cultural & political dynamics in risk communication |
|  |  | C | OPERATIONAL COMMUNICATION  (any communication that is not with the media and public) | 1. Identify various and appropriate sources of or for information in a disaster or public health emergency 2. Discuss the means of verification of information 3. Describe knowledge transfer and information sharing processes for DRM 4. Engage and communicate risks effectively with different stakeholders | 1. Sources of information in disaster situations 2. Modes of information dissemination in DRM 3. Knowledge management in DRM |
| 4 | Demonstrate the knowledge of principles of legal, human rights and ethics in dealing with DRM | A | ETHICS | 1. Discuss key principles of ethics 2. Define ethics in relation to DRM 3. Discuss ethical issues likely to be encountered in disasters and public health emergencies | - Principles of ethics in DRM - Ethical issues in health research during humanitarian crises |
|  |  | B | HUMAN RIGHTS | 1. Describe key principles of human rights 2. Discuss the concept of ‘Right to health’ 3. Discuss the Sphere standards and humanitarian charter | - Principles of human rights & right to health - Humanitarian principles and code of conduct in humanitarian settings (Sphere standards) - Human rights charter |
|  |  | C | International Humanitarian Law | 1. Describe key principles of International Humanitarian Law 2. Discuss legal and regulatory issues likely to be encountered in disasters and public health emergencies 3. Describe legal statutes related to healthcare delivery that may be activated after the declaration of disasters and public health emergencies | - International Humanitarian Law |
|  |  | D | International Health Regulations | 1. Discuss International Health Regulations (IHR) in relations to DRM | 1. International Health Regulation and its application to DRM |
| 5 | Demonstrate ability to identify, mobilise and manage resources | A | RESOURCE MOBILIZATION | 1. Define the concept of resource mobilization and accountability 2. Identify funding mechanisms for DRM and potential sources of resources 3. Discuss the tools and techniques of resource mobilization and accountability 4. Demonstrate proposal development skills | - Basics of resource mobilization - Humanitarian/disaster funding frameworks: Central Emergency Response Fund, Flash appeal, CAP - Basics of proposal development Financial mobilization and accountability |
| 6 | Demonstrate the ability apply logistics management | A | LOGISTICS MANAGEMENT | 1. Describe key principles of logistics management in DRM (tools for logistic management) 2. Discuss the roles and importance of health personnel in logistics systems for emergencies 3. Discuss systems and processes for managing supplies and logistics in emergencies 4. Discuss the challenges and constraints facing the health sector in managing supplies and logistics In emergencies 5. Use communications technology and IT effectively | 1. Principles of logistics 2. Logistics management tools, methods and processes 3. Essentials of Logistics Management Information system in emergency 4. Basics of fleet management 5. Essentials on communication equipment and IT support 6. Basics of field base set up and base management |
| 7 | Demonstrate the ability to apply measures of safety and security | A | BASIC SECURITY IN THE FIELD | 1. Understand key principles of personal security and safety in the field 2. Apply safety measures for site security 3. Apply safety measures for response personnel | - Basics of personal security and safety in the field - Essential of site security - Essentials safety measures for response personnel - Minimum Operating Security Standards (MOSS) |
|  |  | B | PROTECTION AND  FAMILY SAFETY | 1. Apply safety measures for personal and family security | Essential of Security and Safety at home |
| 8 | Demonstrate effective Leadership, teamwork and management skills required for DRM | A | Principles of leadership and management | 1. Define key concepts and principles of leadership, management and coordination | 1. Basics of leadership, management and coordination |
|  |  | B | LEADERSHIP | 1. Describe leadership characteristics and styles 2. Describe the characteristics of effective leadership in the context of health emergency management | 1. Leadership characteristics 2. Leadership styles 3. Effective leadership skills and roles in context of DRM |
|  |  | C | MANAGEMENT | 1. Discuss principles of effective management 2. Identify the key tools and processes which enable health managers to manage DRM programmes | 1. Management functions 2. Management keys tools and process 3. Management challenges in Humanitarian & Disaster situations |
|  |  | D | COORDINATION | 1. Define effective coordination 2. Explain the importance of coordination in DRM 3. Discuss national and international humanitarian coordination systems and tools | 1. Meaning of coordination 2. Key principles of effective coordination and outputs 3. National and International humanitarian coordination system and tools 4. Cluster approach |
| 9 | Demonstration ability to effectively perform monitoring and evaluation. | A | KEY PRINCIPLES | 1. Explain basic principles of M&E 2. Describe M&E frameworks for health programmes 3. Discuss the core contents of M&E plan | 1. Principle of M&E 2. Monitoring and evaluation frameworks 3. Basics of M&E plans |
|  |  | B | MONITORING | 1. Apply monitoring frameworks in all the phases of a disaster and public health emergency 2. Describe methodologies for monitoring morbidity, mortality and health service delivery following disasters | 1. Methods of monitoring in the context of DRM 2. Monitoring tools, health assessments and surveys (mortality surveys) 3. Public health risks monitoring and consequences |
|  |  | C | EVALUATION | 1. Discuss methods of evaluation in the context of DRM 2. Apply evaluation frameworks in all the phases of a disaster and public health emergency | 1. Methods and tools of evaluation 2. Principles of joint evaluation 3. Essential Skills in DRM intervention and program evaluation |

**Pre-disaster – Emergency Preparedness**

|  | **Core competencies** |  | **SESSIONS** | **Learning objectives** | **Main points** |
| --- | --- | --- | --- | --- | --- |
| 10 | Demonstrate the ability to conduct capacity assessments | A | KEY PRINCIPLES | 1. Describe the key principles of capacity assessment 2. Discuss the types of assessments, their methodologies and applications | 1. Concepts and principles of capacity assessment |
|  |  | B | RISK ASSESSMENTS | 1. Demonstrate the ability to conduct risk assessments vulnerability, risk assessment and mapping (VRAM) 2. Apply the result of the risk assessment to improve disaster risk management | 1. Definition of basic terms in disaster risk assessments 2. Basics risk assessments process & methodologies 3. Essentials of VRAM and Hospital safety Index assessments |
|  |  | C | NEEDS ASSESSMENTS | 1. Demonstrate the ability to conduct needs assessments 2. Apply the result of the need assessment to improve disaster risk management | 1. Definitions of needs assessments 2. Needs assessment processes and methodologies 3. Communicating Needs assessment results to decision-makers |
| 11 | Demonstrate the ability to plan and implement preventive and mitigation activities | A | KEY PRINCIPLES | 1. Define key concepts including disaster prevention, mitigation, risk reduction and disaster risk management 2. Discuss national and global frameworks on Disaster risk reduction 3. Discuss Primary Health Care (PHC) approaches and its application to DRM | - Key components of Hyogo Framework for Action in relation public health - Basics of DRR and mitigation steps - Essentials of PHC and its application to DRM |
|  |  | B | RISK REDUCTION | 1. Identify measures to reduce risks at community (eg PHC), Health facilities and national level 2. Develop disaster prevention and mitigation strategy (frameworks) for the health sector | 1. Definition of concepts 2. Basics of risk reduction at community level 3. Highlights of national risk reduction planning frameworks 4. Essential of risk reduction to the health care system (facilities and personnel) |
|  |  | C | MITIGATION | 1. Describe the components of structural and non-structural adaptation for reduction and mitigation 2. Be able to collaborate and engage across multiple sectors and disciplines | 1. Overview of DRM country capacity analysis, health facility safety and community resilience. |
| 12 | Demonstrates the ability to plan and implement emergency preparedness at community and health facility levels | A | KEY PRINCIPLES | 1. Describe the key principles of disaster preparedness 2. Discuss key components of emergency preparedness strategies at the community and health facility levels 3. Explain the importance of educating communities for preparedness for public health emergencies and disasters | - Definition of concepts - Key principles of disaster preparedness - Essentials of health facility and community preparedness |
|  |  | B | PLANNING | 1. Describe the process of emergency planning 2. Explain project planning cycle and key principles of planning 3. Explain the DRM cycle and elements of planning during the various phases 4. Describe the process and application of objective-based planning to emergency preparedness | 1. Defining of emergency planning 2. Basics of emergency planning in the context of health care facilities 3. Preparation of hospital emergency response plan 4. Essentials contingency planning,   Hospital and health facility planning and business continuity planning/continuity of operations |
|  |  | C | EARLY WARNING | 1. Describe the key principles of early warning systems 2. Discuss the main components an early warning system for disasters and public health emergencies | - Definition of Early warning - Key components, methods and tools of early warning in disaster and public health - Data sources and challenges of instituting early warning system |
|  |  | D | SURGE CAPACITY | 1. Describe the key principles of surge capacity 2. Describe the role of key players in surge capacity | 1. Definition of surge capacity 2. Key players in surge capacity 3. Essentials of Mass casualty management |
|  |  | E | TRAINING | 1. Able to conduct a DRM training needs assessment | - Description of DRM training needs assessment - DRM Training needs assessment tools, methods and processes - Report writing and Dissemination of training needs assessment reports |
|  |  | F | EXERCISE MANAGEMENT | 1. Explain the key principles of exercise management 2. Explain the purposes of using drills and exercises for emergency preparedness (testing of plans) 3. Design, conduct, evaluate and use outcomes of simulation exercises to improve preparedness and response | 1. Definition of exercise management 2. Key principles of exercise management 3. Essentials of simulation exercises 4. Simulation planning for testing of national multi-sectoral preparedness and response plans |

**Disaster Phase**

|  | **Individual competencies** |  | **TOPICS /session** | **session learning objectives** | **Main points** |
| --- | --- | --- | --- | --- | --- |
| 13 | Demonstrate ability to apply DRM principles and practices for the health response to disasters and public health emergencies | A | KEY PRINCIPLES | 1. Describe the key principles of disaster response 2. Discuss emerging risks and threats to health during the response phase 3. Identify common causes of morbidity and mortality during response phase 4. Apply emergency SOPs | 1. Key principles of disaster response 2. Health risks and threats during the emergency response phase 3. Essentials of morbidity and mortality prevention during response phase 4. Basics of Emergency SOPs |
|  |  | B | HEALTH ASSESSMENT | 1. Describe the various types of assessment during the response phase 2. Discuss components of initial rapid health needs assessment 3. Describe the methodology of initial rapid health needs assessment 4. Utilize findings of health needs assessments for field actions | 1. Principles of rapid health assessments 2. Types of and role of health workers in health risk assessment 3. Key components, tools, process and methods for conducting rapid health assessment 4. Benefits and challenges associated with health assessment processes, methods and technologies 5. Communicating rapid health assessment results to decision-makers |
|  |  | C | MCM /EMS | 1. Describe the key principles Mass Casualty Management 2. Describe the components of an Emergency Medical Services (EMS) 3. Apply key principles of triage | 1. Definition of mass casualty management 2. Essential skills in mass casualty management 3. Components of emergency medical services 4. Basics of triage |
|  |  | D | INCIDENT MANAGEMENT | 1. Apply the key principles of incident management 2. Describe the components of incident command and control systems 3. Describe the organizational structure of an emergency operations Centre | 1. Definition of incident management 2. Core components of an incident management system 3. Main functions of an incident management system 4. Function of Incident Command Post, Emergency Operations Center and the Emergency Coordination Center 5. roles of a health emergency manager in response coordination and incident management |
|  |  | E | PUBLIC HEALTH PROGRAMMES IN EMERGENCIES | 1. Discuss essential health care interventions and delivery strategy in emergencies 2. Demonstrate an understanding of public health preventive measures during an emergency 3. Describe common interventions to reduce morbidity and mortality during the acute phase of an emergency | - Essential health care services in emergencies - Health service delivery strategy in emergency - Essential public health preventive measures |
|  |  | F | PLANNING | 1. Explain the importance of early planning for disaster recovery during the response phase | 1. Planning cycle, steps, methods and tools used of the emergency response 2. Mechanism of disaster and emergency planning 3. Roles of health workers in the emergency response planning process |

**Post-disaster**

|  | **core competencies** |  | **SESSIONS** | **session learning objectives** | **Main points** |
| --- | --- | --- | --- | --- | --- |
| 14 | Demonstrate ability to plan and implement health system and population recovery. | A | KEY PRINCIPLES | 1. Define key concepts and principles including Health system recovery, Population recovery, resilience 2. Discuss common causes of morbidity and mortality during the recovery process 3. Discuss the linkage between the recovery and routine development strategies. 4. Analyse emerging risks and threats to health during the recovery phase 5. Describe common causes of morbidity and mortality during the recovery process | 1. Definition of key concepts and principles including Health system recovery, Population recovery, resilience 2. Causes of morbidity and mortality |
|  |  | B | RECOVERY NEEDS ASSESSMENTS | 1. Assess/Analyze health system in post disaster situation 2. Conduct post conflict/disaster population needs assessment | 1. Disrupted health systems analysis 2. Post-Conflict/Disaster Needs Assessments (PCNA and PDNA) |
|  |  | C | RECOVERY STRATEGY AND PLANNING | 1. Develop strategies for increasing the resilience of individuals, communities and health systems affected by disasters 2. Discuss the linkage between the recovery and routine development strategies 3. Analyse processes for health system capacity development and health recovery strategy | - Health system and population recovery strategy and link to development |
|  |  | D | PROGRAMME IMPLEMENTATION | 1. Discuss the interventions required for rehabilitation/reconstruction (physical, psychosocial and health systems) | - Community Resilience Building mechanisms, tools and processes in post conflict/disaster populations - Rehabilitation/Reconstructions of the post conflict/disaster affected health system - Health care services in recovery period |
