# Supplementary material for: What should the African health workforce know about disasters? Proposed competencies for strengthening public health disaster risk management education in Africa
Source: BMC Med Educ. 2018 Apr 2;18:60. doi: 10.1186/s12909-018-1163-9 (PMC5879558; doi:10.1186/s12909-018-1163-9)
Supplement: Supplementary file 2 — Curriculum for basic, intermediate and advanced training in health DRM. (DOCX 38 kb) [file 12909_2018_1163_MOESM2_ESM.docx]

**Additional file 2: Curriculum for basic, intermediate and advanced training in health DRM**

### Basic Course Curriculum

| **Course Title** | **Basic Training in Health Disaster Risk Management** |
| --- | --- |
| **Course duration/Time allotment** | **(Universities will put course credits or appropriate units):** |
| **Course rationale:** | The number of emergencies and disasters recorded in the world continues to rise. For instance in 1980, less than 400 emergencies were recorded, compared to 800 emergencies recorded in 2011. The magnitude of the economic loss resulting from disasters and emergencies is enormous, and has been increasing. In 1980, about USD 50 billion was lost due to emergencies and disasters, whereas in 2011 the loss was USD 180 billion.  In the African region of WHO, 32 of the 47 countries experienced at least one emergency between 2008 and 2012. These emergencies affected more than 100 million people. It has been noted that throughout all these emergencies and disasters, there were gaps in managing the emergencies at all levels (community, sub-regional and national levels). The gaps identified included lack of relevant policies, laws, legislation, strategies and capacities to guide the government and in particular the health sector interventions for DRM. There is need to address this gap. This course aims to address some of the gaps by providing a standardized curriculum for health workers and other partners in health DRM, to complement the existing curricula currently being used by the different training Institutions. |
| **Overall purpose:** | The overall purpose of the basic course is to contribute to reducing loss (social, environmental, economic) as a result of disaster, by building the capacity of health workers and partners implementing Health DRM activities, in basic knowledge and skills of health DRM |
| **Course objectives:** | The course will enable participants to:   - - Define and apply basic knowledge about DRM to fulfil their role effectively in DRM   - Impart knowledge, develop skills and promote the right for contribution to building community resilience |
| **Target participants** | This course is recommended for   1. Health worker at the district level/or equivalent. 2. Staff of humanitarian agencies 3. Personnel who contribute to Health DRM from other sectors 4. Persons interested in following a track in DRM as a carrier (academic, professional) |
| **Admission criteria** | Persons eligible for this course must have at least a basic certificate/diploma (nursing, paramedical and/or medicine/dentistry, environmental health, veterinary science or social sciences) |
| **Competencies addressed in this course** | 1. Demonstrate knowledge of public health principles and practices for Disaster Risk Management 2. Demonstrate knowledge of basic epidemiological methods and data management 3. Demonstrate the ability to communicate effectively in DRM 4. Demonstrate the knowledge of principles of legal considerations, human rights and ethics in dealing with DRM 5. Demonstrate the ability to apply measures of safety and security 6. Demonstrate effective leadership, teamwork and management skills required for DRM (only key principles) 7. Demonstrate knowledge about the monitoring and evaluation cycle (only key principles) 8. Demonstrate the ability to conduct capacity assessments (only key principles) 9. Demonstrate the ability to plan and implement preventive and mitigation activities (only key principles) 10. Demonstrate the ability to plan and implement emergency preparedness at community and health facility levels (only key principles) 11. Demonstrate ability to apply DRM principles and practices for the health response to disasters and public health emergencies (only key principles) 12. Demonstrate ability to plan and implement health system and population recovery (only key principles) |
| **Course teaching/**  **Delivery method** | 1. Lectures and case studies 2. Group work 3. Video shows 4. Individual assignments |
| **Resources** | 1. Human resources (from various area of expertise) 2. Teaching space 3. Computers 4. Overhead projectors 5. Video 6. Stationery (pens, paper, masking tape etc) |
| **Evaluation** | The participants will be evaluated through   - - Continuous assessments: learning will be measured through observation, written tasks (at the end of each session, each day, one week),   - Final assessments: through tests (essay, pre and post-test)   The course evaluation will be done by the participants at the end of the training and recommendation made. |
| **Course award** | - Upon successful completion, the participants will be awarded a certificate in basic Health DRM. |
| **Course management and oversight** | In order to ensure quality and standards, the following has been put in place;   1. International quality assurance:    1. WHO/AFRO (guided by the WHO/AFRO regional strategy, and peer review) 2. National council/board for Higher Education:    1. Accreditation of course in collaboration with Ministry of Education, WHO (formal partnership with national institution/individual university/other institution/existing country system) 3. Accredited training providers    1. Training Institutions   **Note**: Verification of an accredited institution will be done by the Ministry of Education, WHO and professional Councils. |
| **Course Director/**  **Managing facilitator** | **To be decided by teaching institution** |

###

### Intermediate Course Curriculum

| **Course Title** | **Intermediate Training in Health Disaster Risk Management** | |
| --- | --- | --- |
| **Course duration/Time allotment** | **(Universities will put course credits or appropriate units):** | |
| **Course rationale:** | The number of emergencies and disasters recorded in the world continues to rise. For instance in 1980, less than 400 emergencies were recorded, compared to 800 emergencies recorded in 2011. The magnitude of the economic loss resulting from disasters and emergencies is enormous, and has been increasing. In 1980, about USD 50 billion was lost due to emergencies and disasters, whereas in 2011 the loss was USD 180 billion.  In WHO African region 32 of the 47 countries experienced at least one emergency between 2008 and 2012. These emergencies affected more than 100 million people. It has been noted that throughout all these emergencies and disasters, there were gaps in managing the emergencies at all levels (community, sub-regional and national level). The gaps identified included lack of relevant policies, laws, legislations, strategies and capacities to guide the government and in particular the health sector interventions for DRM. There is need to address this gap. This course aims to address major gaps by providing a standardized curriculum for training mid-level managers in health DRM. | |
| **Overall purpose:** | The overall purpose of this intermediate course is to contribute to reducing loss (social, environmental, economic) as a result of disaster by building the capacity of mid-level managers at sub-national levels in knowledge, skills and attitudes needed for addressing health DRM. | |
| **Course objectives:** | The course will enable participants to:   - - Apply knowledge of, and skills in health DRM to fulfil their role as effective sub-national managers in DRM.   - Acquire the knowledge, attitude and skills to contribute to building country resilience | |
| **Target participants** | This course is applicable to mid-level health care personnel working in Health DRM at sub-national and national levels. | |
| **Admission criteria** | This course is applicable to participants with the following qualifications;   1. A minimum of a Bachelor’s degree in health or social sciences, with at least 1 year experience in health DRM or, 2. A basic diploma in health or social work, with the certificate of the basic course in Health DRM, or over 3 years’ experience in DRM. | |
| **Competencies addressed in this course** | **Theme** | **Core Competency** |
|  | **Introduction to DRM** | 1. Demonstrate knowledge of public health principles and practices for Disaster Risk Management 2. Demonstrate knowledge of basic epidemiological methods and data management 3. Demonstrate the ability to communicate effectively in DRM 4. Demonstrate the knowledge of principles of legal considerations, human rights and ethics in dealing with DRM |
|  | **Operational Effectiveness** | 1. Demonstrate ability to identify, mobilise and manage resources 2. Demonstrate the ability to apply logistics management 3. Demonstrate the ability to apply measures of safety and security |
|  | **Effective Leadership** | 1. Demonstrate effective leadership, teamwork and management skills required for DRM 2. Demonstrate knowledge about the monitoring and evaluation cycle |
|  | **Preparedness and Risk Reduction** | 1. Demonstrate the ability to conduct capacity assessments 2. Demonstrate the ability to plan and implement preventive and mitigation activities 3. Demonstrate the ability to plan and implement emergency preparedness at community and health facility levels |
|  | **Response and Recovery** | 1. Demonstrate ability to apply DRM principles and practices for the health response to disasters and public health emergencies 2. Demonstrate ability to plan and implement health system and population recovery. |
| **Course teaching/**  **Delivery method** | - Pre-course work - Lectures (in class and/or on line) and case studies - Group work - Practical simulations | |
| **Resources** | - Human resources - Teaching space - Computers - Overhead projectors - Video - Stationery (pens, paper, masking tape, etc.) | |
| **Evaluation** | The participants will be evaluated through:   - - Continuous assessments: Learning will be measured through observation, written tasks (at the end of each session, each day, one week),   - Final assessments: through tests (essay, pre and post-test)   - The course evaluation will be done by the participants at the end of the training and recommendation made | |
| **Course award** | Upon successful completion, the participants will be awarded a certificate in intermediate Health DRM | |
| **Course management and oversight** | 1. International quality assurance:    1. WHO/AFRO (guided by the WHO/AFRO regional strategy and peer review) 2. National council/board for Higher Education:    1. Accreditation of course in collaboration with Ministry of Education (MoE), WHO (formal partnership with national institution/individual university/other institution/existing country system)    2. National quality assurance of course 3. Accredited training providers    1. Training Institutions   **Note:** Verification of accredited institution will be done by MoE, WHO, National Professional Councils. | |
| **Course director/Managing facilitator** | **To be decided by teaching institution** | |

### Advanced Course Curriculum

| **Course Title** | **Advanced Training in Health Disaster Risk Management** |
| --- | --- |
| **Course duration/Time allotment** | **Course duration/Time allotment (Universities will put course credits or appropriate units)** |
| **Course rationale:** | The number of emergencies and disasters recorded in the world continues to rise. For instance in 1980, less than 400 emergencies were recorded, compared to 800 emergencies recorded in 2011. The magnitude of the economic loss resulting from disasters and emergencies is enormous, and has been increasing. In 1980, about USD 50 billion was lost due to emergencies and disasters, whereas in 2011 the loss was USD 180 billion.  In WHO African region 32 of the 47 countries experienced at least one emergency between 2008 and 2012. These emergencies affected more than 100 million people. It has been noted that throughout all these emergencies and disasters, there were gaps in managing the emergencies at all levels (community, sub-regional and national level). The gaps identified included lack of relevant policies, laws, legislations, strategies and capacities to guide the government and in particular the health sector interventions for DRM. There is need to address this gap. This course aims to address some of the management and leadership gaps at regional and national levels, by providing a standardized curriculum for training leaders and managers in health DRM. |
| **Overall purpose:** | The overall purpose of this advanced course is to contribute to reducing loss (social, environmental, economic) as a result of disasters, by building the capacity of leaders and managers at national level in Health DRM activities, with advanced knowledge and skills in health DRM |
| **Course objectives:** | The course will enable participants to:   - Apply advanced management and leadership knowledge and skills about DRM to fulfil their role at national levels effectively - Have the knowledge, attitude and skills to contribute to building country resilience |
| **Target participants** | This course is applicable to all personnel working in Health DRM at regional and national levels, and those intending to lead and manage Health DRM at national level. |
| **Admission criteria** | This course is applicable to participants with the following qualifications;   - A basic or Master’s degree in health or social sciences, with at least 3 years’ experience in health DRM, PLUS - The certificate in the Intermediate course in Health DRM. - Attendance of the intermediate course is a prerequisite for being selected for this course |
| **Competencies addressed in this course** | This course mainly involves an internship period of about 8 weeks, during which participants will undergo practical field placements. One week will spent on orientation and briefing by the training institution, 6 weeks on field work, and 1 week for report writing, debriefing and oral defence of the report. The internship exercise involves application of the knowledge, attitude and skills related to the DRM cycle. Therefore, the intern will rotate through either:   - An emergency situation, preferably at the local level - Field work on preparedness for a disaster   Academic and field supervisors should be people experienced in health DRM i.e. national authorities and international agencies. Selection of internship site/organization should be locally negotiated by the training institution. There should be some level of interaction between both supervisors so as to ensure that the intern obtains the appropriate exposure  **Tasks to be accomplished during placement**  **Disaster/public health emergency outputs**  1. A rapid assessment conducted during a disaster and/ or outbreak investigation during a public health emergency:   1. Collect and analyse relevant data 2. Write detailed report with clear recommendations for short and long term response   2. Outcome proposals produced following the assessment:   1. Prioritize key interventions 2. Develop a project proposal for comprehensive disaster/epidemic response 3. Develop key fundraising documents such as FLASH/CERF appeal   3. Participation in the design, implementation, supervision and monitoring of key response interventions such as:   1. Disease surveillance 2. Programme implementation e.g. immunization activities, service delivery etc. 3. Coordination of field partners during the emergency phase   4. Production of a strategic outline document to guide the recovery phase  5. A Debrief with field and academic supervisors on completion of placement. Debrief will require a comprehensive technical report of the field placement  **Preparedness outputs**   1. Risk analysis for potential disaster/public health emergencies using objective criteria 2. A short strategy on health disaster risk reduction developed 3. Collaboration with local authorities, to design appropriate preparedness and response programmes:    1. Early warning systems    2. Contingency planning and simulation    3. Design and implementation of response activities    4. Conduct appropriate training 4. A strategic outline document for the response phase produced 5. Debrief with field and academic supervisors on completion of placement.. Debrief will require a comprehensive technical report of the field placement |
| **Course teaching/**  **Delivery method** | - pre-course work - field work/practical simulations - Field placement |
| **Resources** | - Human resources (of relevant disciplines) - Teaching space - Computers - Overhead projectors - Video - Stationery (pens, paper, masking tape etc) - Field placement sites - Transport to field placement sites |
| **Evaluation** | The assessment will be done in 3 main ways:   - Observation and recommendation of the field supervisors (20%) - Written field report (50%) - Verbal report – defence of the report (30%).   The course evaluation will be done by the participants at the end of the training and recommendations made. |
| **Course award** | Upon successful completion, the participants will be awarded the advanced certificate in Health DRM. |
| **Course management and oversight** | 1. International quality assurance:    1. WHO/AFRO (guided by the WHO/AFRO regional strategy, and peer review) 2. National council/board for Higher Education:    1. Accreditation of course in collaboration with Ministry of Education (MoE), WHO (formal partnership with national institution/individual university/other institution/existing country system)    2. National quality assurance of course 3. Accredited training providers    1. Training Institutions   Note: Verification of accredited institution will be done by MoE, WHO, National Professional Councils. |
| **Course director/Managing facilitator** | **To be decided by teaching institution** |
