# Supplementary material for: What should the African health workforce know about disasters? Proposed competencies for strengthening public health disaster risk management education in Africa
Source: BMC Med Educ. 2018 Apr 2;18:60. doi: 10.1186/s12909-018-1163-9 (PMC5879558; doi:10.1186/s12909-018-1163-9)
Supplement: Supplementary file 3 — Proposed public health DRM training modules. (DOCX 23 kb) [file 12909_2018_1163_MOESM3_ESM.docx]

**Additional file 3: Proposed public health DRM training modules**

| **Themes** | **Unit** | **Session** | **Course Content** | | **No of Hours*** | | **Module & Units/Sessions in Module** | | **No of Credit of Module** |
| --- | --- | --- | --- | --- | --- | --- | --- | --- | --- |
|  |  |  | **Basic** | **Intermediate** | **Basic** | **Intermediate** | **Basic** | **Intermediate** |  |
| **1: Introduction to DRM** | 1. Demonstrate knowledge of public health principles and practices for Disaster Risk Management | Disaster risk management concepts | X | X | 8 | 8 | **Module 1**  **(48 hours)** | **Module 1**  **(58 hours)** |  |
|  |  | Public health consequences of disasters | X | X |  |  |  |  |  |
|  |  | Context: political, social and economic environment | X | X |  |  |  |  |  |
|  | 1. Demonstrate knowledge of basic epidemiological methods and data management | Basic epidemiology | X | X | 8 | 16 |  |  |  |
|  |  | Data analysis and management | X | X |  |  |  |  |  |
|  | 1. Demonstrate the ability to communicate effectively in DRM | Key principles | X | X | 4 | 4 |  |  |  |
|  |  | Risk communication | X | X |  |  |  |  |  |
|  |  | Operational communication  (any communication that is not with the media and public) |  | X |  |  |  |  |  |
|  | 1. Demonstrate the knowledge of principles of legal, human rights and ethics in dealing with DRM | Ethics | X | X | 6 | 6 |  |  |  |
|  |  | Human rights | X | X |  |  |  |  |  |
|  |  | International humanitarian law | X | X |  |  |  |  |  |
|  |  | International health regulations |  |  |  |  |  |  |  |
| **2: Operational Effectiveness** | 1. Demonstrate ability to identify, mobilise and manage resources | Resource mobilization |  | X | 0 | 4 |  |  |  |
|  | 1. Demonstrate the ability to apply logistics management | Logistics management |  | X | 0 | 4 |  |  |  |
|  | 1. Demonstrate the ability to apply measures of safety and security | Basic security in the field | X | X | 4 | 4 |  |  |  |
|  |  | Protection and family safety | X | X |  |  |  |  |  |
| **3: Effective leadership** | 1. Demonstrate effective leadership, teamwork and management skills required for DRM | Principles of leadership, management and coordination | X | X | 2 | 6 |  |  |  |
|  |  | Leadership |  | X |  |  |  |  |  |
|  |  | Management |  | X |  |  |  |  |  |
|  |  | Coordination |  | X |  |  |  |  |  |
|  | 1. Demonstration knowledge about the monitoring and evaluation cycle | Key principles | X | X | 2 | 6 |  |  |  |
|  |  | Monitoring |  | X |  |  |  |  |  |
|  |  | Evaluation |  | X |  |  |  |  |  |
| **4: Preparedness and Risk Reduction** | 1. Demonstrate the ability to conduct capacity assessments | Key principles | X | X | 2 | 16 |  | **Module 2**  **(36 hours)** |  |
|  |  | Risk assessments |  | X |  |  |  |  |  |
|  |  | Needs assessments |  | X |  |  |  |  |  |
|  | 1. Demonstrate the ability to plan and implement preventive and mitigation activities | Key principles | X | X | 2 | 4 |  |  |  |
|  |  | Risk reduction |  | X |  |  |  |  |  |
|  |  | Mitigation |  | X |  |  |  |  |  |
|  | 1. Demonstrates the ability to plan and implement emergency preparedness at community and health facility levels | Key principles | X | X | 2 | 16 |  |  |  |
|  |  | Planning |  | X |  |  |  |  |  |
|  |  | Early warning |  | X |  |  |  |  |  |
|  |  | Surge capacity |  | X |  |  |  |  |  |
|  |  | Training |  | X |  |  |  |  |  |
|  |  | Exercise management |  | X |  |  |  |  |  |
| **5: Response and recovery** | 1. Demonstrate ability to apply DRM principles and practices for the health response to disasters and public health emergencies | Key principles | X | X | 6 | 16 |  | **Module 3**  **(32 hours)** |  |
|  |  | Health assessment |  | X |  |  |  |  |  |
|  |  | MCM /EMS | X | X |  |  |  |  |  |
|  |  | Incident management |  | X |  |  |  |  |  |
|  |  | Public health programmes in emergencies |  | X |  |  |  |  |  |
|  |  | Planning |  | X |  |  |  |  |  |
|  | 1. Demonstrate ability to plan and implement health system and population recovery. | Key principle | X | X | 2 | 16 |  |  |  |
|  |  | Recovery needs assessments |  | X |  |  |  |  |  |
|  |  | Recovery strategy and planning |  | X |  |  |  |  |  |
|  |  | Programme implementation |  | X |  |  |  |  |  |
|  |  | Integrated Simulation |  |  |  | 8 |  |  |  |
| **Total no of hours** |  |  |  |  | **48** | **134** |  |  |  |
| **Number of modules** |  |  |  |  | **1** | **3** |  |  |  |

**This includes number of hours spent on lectures, practical sessions, assignments, assessments and self-reading*
